# Chlorinated Pool Attendance, Atopy, and the Risk of Asthma during Childhood

**DOI:** 10.1289/ehp.8461

**Published:** 2006-06-08

**Authors:** Alfred Bernard, Sylviane Carbonnelle, Claire de Burbure, Olivier Michel, Marc Nickmilder

**Affiliations:** 1 Department of Public Health, Catholic University of Louvain, Brussels, Belgium; 2 Clinics of Allergology and Respiratory Diseases, Free University of Brussels, Brussels, Belgium

**Keywords:** aeroallergens, atopy, childhood asthma, chlorine, exercise-induced asthma, exhaled nitric oxide, nitrogen trichloride, swimming pool, total IgE, trichloramine

## Abstract

The pool chlorine hypothesis postulates that the rise in childhood asthma in the developed world could result at least partly from the increasing exposure of children to toxic gases and aerosols contaminating the air of indoor chlorinated pools. To further assess this hypothesis, we explored the relationships between childhood asthma, atopy, and cumulated pool attendance (CPA). We studied 341 schoolchildren 10–13 years of age who attended at a variable rate the same public pool in Brussels (trichloramine in air, 0.3–0.5 mg/m^3^). Examination of the children included a questionnaire, an exercise-induced bronchoconstriction (EIB) test, and the measurement of exhaled nitric oxide (eNO) and total and aeroallergen-specific serum IgE. CPA by children (range, 0–1,818 hr) emerged among the most consistent predictors of asthma (doctor diagnosed or screened with the EIB test) and of elevated eNO, ranking immediately after atopy and family history of asthma or hay fever. Although the risk of elevated eNO increased with CPA [odds ratio (OR) = 1.30; 95% confidence interval (CI), 1.10–1.43] independently of total or specific serum IgE, the probability of developing asthma increased with CPA only in children with serum IgE > 100 kIU/L (OR for each 100-hr increase in CPA = 1.79; 95% CI, 1.07–2.72). All these effects were dose related and most strongly linked to pool attendance before 6–7 years of age. Use of indoor chlorinated pools especially by young children interacts with atopic status to promote the development of childhood asthma. These findings further support the hypothesis implicating pool chlorine in the rise of childhood asthma in industrialized countries.

The prevalence of allergic diseases such as atopic asthma and eczema has dramatically increased in the developed world over the past decades. In the United States, as in most industrialized countries, asthma has become the most common chronic childhood disease. Intriguingly, countries the most affected by this rise are English-speaking countries such as the United Kingdom, Ireland, Australia, and New Zealand, where prevalence rates of childhood asthma are up to 10-fold higher than in most southern and eastern countries (American Lung Association 2004; Johnsson et al. 2002).

The causes of both the rise and international disparities in childhood asthma prevalence are largely unknown. Given the rapidity of the rise, genetic factors alone cannot explain this phenomenon, and research has thus turned its attention to changes in environment and, more recently, in lifestyle. One of the hypotheses, which generated most interest, is the “hygiene hypothesis” postulating that the rise of allergic diseases in industrialized countries could be caused by the declining exposure of children to infections during early infancy (Strachan 1989). Although the T-helper types 1–2 (T_H_1–T_H_2) paradigm provides a convincing mechanistic support to the hygiene hypothesis (McGeady 2004), epidemiologic studies still generate conflicting results and, despite intense research, have not yet succeeded in causally linking the asthma rise to specific risk factors that might serve as a basis for preventive actions (Kramer et al. 2004; Liu and Murphy 2003; Sheikh et al. 2003; Strachan 2000). The hygiene hypothesis has also been challenged by some recent experimental data, which suggest that, if at all protective, the effects of infections could be limited to a handful of pathogens (e.g., parasites) and operate only within narrow windows of opportunity during early life (Umetsu 2004).

Recently, a hitherto unsuspected factor, so deeply rooted in our hygienic Western way of life that it had never been investigated, has come to light with the finding that the attendance at indoor chlorinated pools correlated with lung epithelium hyperpermeability and asthma prevalence in children (Bernard et al. 2003). This finding led to the pool chlorine hypothesis, proposing that the increasing attendance at indoor chlorinated pools by increasingly younger children could be implicated in the childhood asthma rise, most probably by interacting with other risk factors (Bernard et al. 2003). The main culprit might be trichloramine (or nitrogen trichloride), an irritant gas released in pool air when chlorine reacts with organic matter brought by swimmers (Massin et al. 1998). Trichloramine has the same irritating potency as chlorine and formaldehyde (Gagnaire et al. 1994) and can cause eye and upper respiratory tract irritation in lifeguards and other pool attendees (Massin et al. 1998). Concentrations of trichloramine in public indoor pools vary greatly depending on pool occupancy and ventilation. Levels of trichloramine typically fluctuate between 0.2 and 0.9 mg/m^3^, with mean values around 0.5 mg/m^3^ (Bernard et al. 2003; Massin et al. 1998), making this gas one of the most concentrated air pollutants to which children of industrialized countries are regularly exposed (mean concentrations of other indoor or outdoor air pollutants seldom exceed 0.3 mg/m^3^; World Health Organization 2000). For many years, trichloramine was believed to be an upper respiratory tract irritant only, clearly a wrong premise for this water-insoluble irritant that can cause asthma in lifeguards (Thickett et al. 2002) and epithelial damage in the deep lung of rodents and recreational swimmers (Bernard et al. 2003; Carbonnelle et al. 2002; Lagerkvist et al. 2004).

In this study focused on schoolchildren, we examined the relationships between asthma, atopy, and chlorinated pool attendance by using different outcome measures and studying age-related variations in exposure and susceptibility.

## Materials and Methods

### Study population

Children were recruited from 10 primary schools located within the same area in southwestern Brussels. After an information session organized in each classroom, a questionnaire and an informed consent document were distributed to the fifth-and sixth-grade schoolchildren. Of about 800 children who received these documents, 527 returned both the questionnaire and the written informed consent, and 365 had the agreement of their parents to participate in all tests including blood analyses. We excluded 10 children who could not provide blood and another 14 because of incomplete information provided on the questionnaire. The final studied population thus included 341 children (172 boys and 169 girls) with a mean (± SD) age of 11.5 ± 0.70 years (range, 10–13 years). The study protocol was approved by the ethics committee of the Faculty of Medicine of the Catholic University of Louvain and complies with all applicable requirements of the United States and/or international regulations. Children were examined only with the written permission of their parents or of the person responsible for them.

Comparison between participants (*n* = 341) and nonparticipants (*n* = 162) who returned the questionnaire did not reveal any significant difference in prevalence of doctor-diagnosed asthma and respiratory diseases or symptoms (e.g., wheezing) or in ethnicity, parental asthma, hay fever or eczema, breast-feeding, regular use of household chlorine bleach, or exposure to environmental tobacco smoke at home or to pets. There were also no significant differences between participants and nonparticipants in the proportions of children having swum as babies (i.e., < 2 years of age), having a backyard pool, being a member of a swimming club, or regularly practicing a sport other than swimming. The only differences that were noted between participants and nonparticipants concerned maternal smoking during the pregnancy (18.6 vs. 11.5%, respectively; *p* = 0.049) and child care attendance (53.4 vs. 40.0%; *p* = 0.007).

### Questionnaire

Parents were asked to complete a questionnaire inquiring, among other items, about family history of allergic diseases, recurrent infectious diseases, asthma and allergic diseases, respiratory symptoms (wheezing, cough, chest tightness, and shortness of breath during the previous 12 months), and exposures or care during early life (maternal smoking during pregnancy, birth weight, breast-feeding, child care attendance before 2 years of age). There were also several questions about lifestyle or environmental factors likely to be involved in the development of respiratory or allergic diseases such as number of siblings, housing density, sporting activity, living with pets at home, exposure to environmental tobacco smoke, use of household chlorine bleach, house with double-glazed windows, mold on child’s bedroom walls, and living in a rural or urban area. The questionnaire also included questions specifically developed to calculate for each year since birth the total time each child spent weekly in an indoor chlorinated pool with the school or their parents or as a sporting activity with a club. Parents were also asked about the existence of an outdoor or indoor pool at home and whether their child had regularly attended a pool before 2 years of age (swimming baby).

### Swimming pool attendance

Swimming pool attendance was a compulsory activity in 7 of the 10 schools studied. In five schools, children attended the pool fortnightly from the third year of kindergarten onward, whereas in the two others they attended the pool from the first year of kindergarten, fortnightly in one school and weekly in the other. Most children also regularly attended a pool either as a recreational activity with their parents or for sport swimming as members of a swimming club. Forty children had regularly swum as babies, and 15 others had a pool at home. About 90% of the children attended the same indoor public pool, which had two pools disinfected with sodium hypochlorite, a small one for younger children and a large one that children attended only when they could swim. The levels of active and combined chlorine in water were within the limits recommended in Belgium (< 1.5 and < 2 mg/L, respectively). The levels of trichloramine in air measured between 2001 and 2003 varied from 0.25 to 0.48 mg/m^3^. The two other pools attended by about 10% of children had similar characteristics, with trichloramine levels in air ranging from 0.26 to 0.54 mg/m^3^.

### Examination of children

All children were examined in the schools between 28 March and 29 May 2002, thus outside main periods of pollination in Belgium. Examinations took place the morning between approximately 0900 hr and 1300 hr to minimize circadian variations. Examination started with the measurement of height and weight and the collection of one blood sample on a dry tube (10 mL). The nitric oxide (NO) concentration in exhaled breath (eNO) was then measured online by chemiluminescence using the NIOX analyzer (Aerocrine AB, Solna, Sweden). The test was performed in compliance with the guidelines of the American Thoracic Society (1999). Children were considered to be positive when having an eNO concentration > 30 ppb. This cutoff level was established as the 95th percentile of the eNO values of children without doctor-diagnosed asthma and a negative exercise-induced bronchoconstriction (EIB) test. Asthma was screened using an EIB test, which consisted in measuring the fall in forced expiratory volume in 1 sec (FEV_1_) after 6 min running with submaximal effort (> 190 heart beats/min) (McFadden and Gilbert 1994). Heart rate was monitored continuously with a polar Electro OY (Kempele, Finland). The test was performed indoors to avoid confounding by weather conditions. Forced vital capacity and FEV_1_ were then measured with a Vitalograph-Compact (Vitalograph Ltd., Buckingham, UK) according to American Thoracic Society (1995) standards. At least three measurements were taken before exercise and three measurements 5 and 10 min after exercise until a minimum of two values differing by < 5% were obtained. The highest value was adopted each time. A reduction in FEV_1_ of 20% or more at 5 or 10 min postexercise was considered a significant EIB. We then calculated total asthma prevalence as the prevalence of children with a positive EIB test plus the prevalence of children who had negative EIB tests but with doctor-diagnosed asthma reported in the questionnaire (ever doctor-diagnosed asthma). We also calculated the prevalence of children with elevated eNO without doctor-diagnosed asthma as well as the prevalence of children having at least one positive outcome, that is, elevated eNO, doctor-diagnosed asthma, and/or a positive EIB test (children with elevated eNO and/or total asthma). Because of time constraints imposed by the schools, skin prick tests could not be performed in addition to all the above tests and were replaced by the assay of allergen-specific immunoglobulin E (IgE), known to provide concordant and equivalent results (Schuetze et al. 1999).

### Total and specific serum IgE

We measured total IgE concentration in serum using the Immulite total IgE kit (DPC, Los Angeles, CA, USA). Aeroallergen-specific serum IgE were first screened using the Immulite AlaTOP allergy screen test detecting IgE against 12 allergens most commonly associated with inhalant allergy (*Dermatophagoides pteronyssinus*, cat epithelium, dog dander, bermuda grass, timothy grass, *Penicillium notatum*, *Alternaria tenuis*, birch, Japanese cedar, common ragweed, English plantain, *Parietaria officinalis*). We also measured separately serum IgE against *D. pteronyssinus*, cat epithelium, dog dander, timothy grass, birch, and *Artemisia vulgaris*. Children were classified as atopic either on the basis of the aeroallergens test when having IgE directed against at least one aeroallergen or on the basis of serum total IgE using as cutoff concentration either 100 or 56 kIU/L, the latter corresponding to the 50th percentile of values measured in our study.

### Data analysis

We used the Student’s *t*-test or the chi-square test to assess statistical significance in bivariate analyses. When necessary, variables were normalized by log-transformation. We used the Mann-Whitney test to compare means of cumulated pool attendance (CPA). Because outcome variables such as doctor-diagnosed asthma, EIB test, eNO test, and respiratory symptoms actually reflect different stages or phenotypes of asthma, likely to be influenced by different predictors, they were analyzed separately or in different combinations. This approach also enabled us to assess the consistency of associations across independent variables while avoiding a distortion of the analysis by a possible greater propensity of children to attend a swimming pool more frequently once asthma had been diagnosed. In a first step, we used logistic-regression models to identify predictors of outcome variables. The following independent variables were tested: maternal and/or paternal history of asthma, maternal and/or paternal history of hay fever, mother and/or father with eczema, total IgE in serum (units of 100 kIU/L), aeroallergen-specific IgE, number of siblings, pets at home since birth, pets at home for < 2 years, housing density (persons/room), chlorine bleach use, passive smoking, breast-feeding, maternal smoking during pregnancy, child care attendance, sport other than swimming, recurrent colds during infancy, and CPA. We checked these independent variables for the absence of multicollinearity by calculating the tolerance and variance inflation factors for each variable (Allison 1999). Second, to test interactions between CPA and atopy, we repeated these logistic regression analyses by separating nonatopic and atopic children using as criterion for atopy either total serum IgE or aeroallergen-specific IgE, or else the combination of these two criteria. Third, with a view to evaluating the influence of the children’s age on their exposure or sensitivity to pool chlorine, we conducted the same logistic regression analyses as above but by testing CPA indices calculated over increasing periods after birth extending to 3–10 years of age. Associations identified by logistic regression analyses were further assessed by comparing the prevalences of asthma (doctor diagnosed or EIB test screened) and elevated eNO in children stratified according to their CPA, examining separately atopics and nonatopics. Only odds ratios (ORs) adjusted for covariates are reported. Significance of dose–response relationships was assessed by a chi-square test for trend.

## Results

As shown in Table 1, 40 children (11.7%) had asthma, either diagnosed by a doctor or screened with the EIB test. The eNO test was positive in 29 children (8.5%), among whom 13 had asthma (44.8%). Most children positive in the eNO test were also positive for aeroallergen-specific IgE (*n* = 26) and had elevated total serum IgE (> 100 kIU/L, *n* = 23). Wheezing was reported by 32 (9.4%) children but was associated with asthma or a positive eNO test in only about half of them (*n* = 17). The prevalences of asthma and wheezing were about 50% higher in boys than in girls. Boys also had significantly higher prevalences of house-mite–specific or cat-specific IgE than did girls, but there were no sex differences in the levels of total serum IgE or of eNO or in the prevalences of pollen- or dog-specific IgE.

As expected, the most statistically significant predictors of doctor-diagnosed asthma, total asthma, and elevated eNO, analyzed separately or in combination, were total and aeroallergen-specific serum IgE and a family history of asthma or hay fever (Table 2). Among tested environmental or lifestyle variables, CPA emerged as one of the most consistent and strongest risk factors. Association with CPA was particularly strong for the risk of elevated eNO (*p* = 0.0027), persisting even after exclusion of children with doctor-diagnosed asthma (*p* = 0.049). Other predictors positively associated with the risk of asthma and/or elevated eNO were housing density, number of siblings, and exposure to pets. No relationship was found between any of the outcomes with child care attendance, environmental tobacco smoke, or maternal smoking during pregnancy (all *p* > 0.30). Asthma risk, interestingly, tended to decrease with the use of chlorine for house cleaning. The risk of elevated eNO was inversely related to body mass index (BMI), child care attendance, and exposure to pets during the preceding 2 years. Although we found no relation with wheezing or shortness of breath (*p* > 0.2), CPA was associated with an increased risk of chest tightness (OR = 1.17; *p* = 0.034) and coughing (OR = 1.11; *p* = 0.069). An even stronger association with CPA was found when combining respiratory symptoms, particularly with the probability of presenting together at least three respiratory symptoms (OR = 1.23; *p* = 0.009). The only other lifestyle variable associated with respiratory symptoms was maternal smoking during pregnancy (with wheezing, OR = 2.75; *p* = 0.041).

To assess interactions between pool attendance and atopy, we compared the ORs for the different outcomes between atopic and nonatopic children by defining atopy on the basis of total IgE or allergen-specific serum IgE. As shown in Table 3, CPA was associated with an increased risk of doctor-diagnosed or total asthma only in children with total serum IgE above the 50th percentile. One of the strongest associations (OR = 1.79; *p* = 0.007) was found for total asthma risk in children with serum IgE > 100 kIU/L, suggesting an approximately 80% increase in asthma risk per 100 hr of CPA. eNO showed a pattern of interaction with CPA that was almost the opposite of that observed with asthma, because the risk of having an elevated eNO was mainly increased in children whose total serum IgE was < 100 kIU/L, the increase remaining significant even below the 50th percentile (Table 3). The associations between eNO and CPA were also remarkably strong (*p*-values down to 0.003) and even persisted when excluding children with a doctor diagnosis of asthma. Most of these interactions between pool attendance and atopy were less significant when atopy was defined on the basis of aeroallergen-specific IgE, and they were not strengthened by the combined use of total and specific IgE. No interaction was found between CPA and atopy in the occurrence of respiratory symptoms.

We ascertained that these pool attendance/atopy interactions were not generated by a particular group of swimmers or the result of a distortion by other risk factors such as family history of asthma or passive exposure to tobacco smoke. Among children with total serum IgE > 100 kIU/L, the association between total asthma and CPA was not weakened by the exclusion of children who were members of a swimming club (OR = 1.89; *p* = 0.0092), who had access to a backyard pool (OR = 1.82; *p* = 0.0072), or who had been swimming babies (OR = 1.95; *p* = 0.0053). Nor were these associations altered by the exclusion of children with a family history of asthma (OR = 1.84; *p* = 0.012) or who were exposed to environmental tobacco smoke (OR = 1.99; *p* = 0.016) or to maternal smoking during pregnancy (OR = 2.03; *p* = 0.022). Similar results were obtained for eNO and other outcomes or by setting the cutoff for total serum IgE at 56 kIU/L, the 50th percentile value (data not shown).

**Figure 1:**
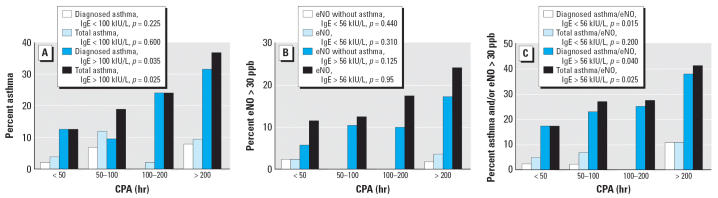
Prevalence of children with doctor-diagnosed or total asthma (*A*), with elevated eNO (*B*), or with doctor-diagnosed or total asthma and/or elevated eNO (*C*) according to the CPA in children with low or high total serum IgE. Total asthma refers to asthma diagnosed by a doctor and/or screened with the EIB test. The cutoff value of 56 kIU/L for total serum IgE corresponds to the 50th percentile of concentrations observed in the studied population. Statistical significance assessed by a chi-square test for trend.

Interactions between pool attendance and atopy were confirmed by the analysis of dose–response relationships (Figure 1). In children with total serum IgE > 100 kIU/L, the prevalence of doctor-diagnosed asthma or of total asthma increased almost linearly with CPA, whereas absolutely no trend was observed below this threshold. Similar dose-related trends were observed with the prevalence of elevated eNO even when excluding children with doctor-diagnosed asthma. When these outcomes were combined, almost linear dose relationships were also found with CPA among children with high concentrations of total serum IgE.

**Figure 2:**
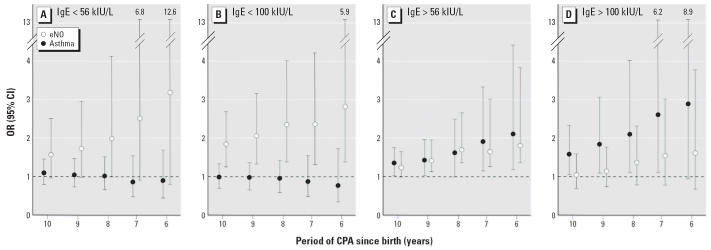
Prevalence of children with high total serum IgE or with aeroallergen-specific serum IgE according to CPA [error bars indicate 95% confidence interval (CI)]. The cutoff value of 56 kIU/L for total serum IgE corresponds to the 50th percentile of concentrations observed in the studied population. Numbers above error bars are upper limits of 95% CIs.

We also assessed the effect of exposure timing on the pool attendance/atopy interaction by calculating the ORs of total asthma or elevated eNO for CPA indices cumulated over an increasing number of years since birth. This analysis was done separately for atopic and nonatopic children using as criterion for atopy a total serum IgE concentration > 56 kIU/L or > 100 kIU/L. Although CPA calculated over any period of time did not increase asthma risk in children with total serum IgE < 56 or < 100 kIU/L, above these two thresholds the ORs gradually raised as CPA was calculated over increasingly younger age groups (Figure 2). We found a similar pattern of increasingly significant ORs for the risk of elevated eNO except that it concerned children with serum IgE < 100 kIU/L as opposed to the pattern observed with total asthma. In these children, the OR for elevated eNO was already significantly higher than 1.0 with pool attendance cumulated up to 3 years of age (OR = 1.20; *p* = 0.027).

Associations between pool attendance and risk of asthma or elevated eNO were not biased by the possibility that children with a medical diagnosis of asthma or living with asthmatic parents had attended swimming pools more frequently. CPA indices calculated over any period of time extending from birth to 10 years of age were indeed statistically not different between children with doctor-diagnosed asthma or parental asthma and children without diagnosis of asthma and history of parental asthma (*p*-values between 0.141 and 0.948). By contrast, among children with serum IgE > 56 kIU/L, the CPA indices were systematically higher in those with total asthma and/or elevated eNO than in those without any of these conditions (*n* = 124), the differences being statistically significant with CPA indices calculated up to 7 (*p* = 0.022), 8 (*p* = 0.011), 9 (*p* = 0.014), or 10 (*p* = 0.041) years of age.

**Figure 3:**
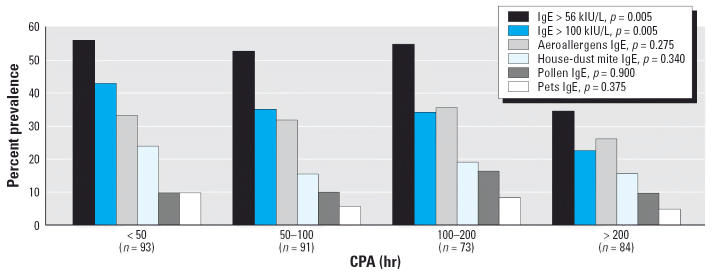
Risk of developing asthma (total asthma, i.e., diagnosed by a doctor or screened with the EIB test) or of having elevated eNO (> 30 ppb) according to total serum IgE level and CPA over increasingly shorter periods of time after birth. The ORs have been adjusted for covariates listed in Table 2. The cutoff value of 56 kIU/L for total serum IgE corresponds to the 50th percentile of concentrations observed in the studied population.

We also checked whether our results could have been distorted by a greater propensity of atopic children to practice swimming than the other children. As shown in Figure 3, the prevalence of children with aeroallergen-specific IgE did not vary significantly with CPA whereas on the contrary the proportion of children with high total serum IgE (> 100 or > 56 kIU/L) decreased with CPA. Logistic regression analysis confirmed these inverse relationships between total serum IgE and CPA (OR for serum IgE > 56 kIU/L 0.85; *p* = 0.014). Other predictors of serum IgE > 56 kIU/L were a history of recurrent ear infections (OR = 0.36; *p* = 0.003), ethnicity (nonwhites vs. whites, OR = 2.35; *p* = 0.002), and number of siblings (OR for each additional sibling = 1.19; *p* = 0.051). Predictors of aeroallergen-specific serum IgE included male sex (OR = 1.77; *p* = 0.023), parental hay fever (OR = 1.95; *p* = 0.010), history of recurrent colds (OR = 2.27; *p* = 0.003), number of siblings (OR for each additional sibling = 1.29; *p* = 0.009), the use of household chlorine bleach (OR = 0.47; *p* = 0.003), and birth weight (OR for each additional 100 g = 0.94; *p* = 0.012).

## Discussion

The present study provides further evidence that regular attendance at indoor chlorinated pools by children is associated with an increased likelihood of developing asthma or of airway inflammation as assessed by the eNO test. Whether these outcomes are tested separately or in combination, CPA ranks as one of the strongest and most consistent predictors immediately after atopy and family history of allergic diseases. The association between pool attendance and asthma was largely the consequence of an interaction with atopic status as assessed on the basis of total serum IgE, which is one of the strongest risk factors for childhood asthma, independently even of allergy (Beeh et al. 2000; Klinnert et al. 2001). Other risk factors of asthma or lung inflammation were variables influenced by housing conditions, particularly siblings, housing density, and pets. Maternal smoking during pregnancy and parental smoking at home were not associated with an increased risk of asthma or elevated eNO. This is at variance with previous studies that have linked childhood asthma to passive exposure to tobacco smoke, especially during fetal life (Gilmour et al. 2006; Johnsson et al. 2002). A possible explanation for this difference is that previous studies were mostly based on larger cohorts of children and thus had a greater statistical power than did our study. These studies have usually examined younger children, which is another possible explanation because the effects of passive smoking on asthma risk are the strongest during early life and progressively diminish with increasing age. Nevertheless, we found that maternal smoking during pregnancy increased the risk of wheezing, an observation consistent with the deleterious effect of *in utero* exposure to tobacco smoke.

Response or selection bias cannot explain these results, which were based on objective outcome measures such as doctor-diagnosed asthma, eNO, and EIB tests. Even assuming a recall or other bias in the parental responses to the questionnaire about their child’s health or pool attendance, it appears unconceivable that this bias could have concerned only children with high serum IgE, generating in this category almost linear dose–response relationships with CPA. The risks of misclassification and response bias were also considerably reduced by the design of our study. We recruited a relatively homogeneous population of schoolchildren from the same area of Brussels and analyzed them using criteria that participants were unaware of when completing the questionnaire (e.g., total or aeroallergen-specific IgE in serum and CPA indices over increasing periods of time after birth). Because the study required blood sampling in schools, we could not achieve a response rate as high as those attained in questionnaire-based studies. However, the comparison of questionnaires filled by participants and by nonparticipants did not reveal any particular bias in outcomes or in major risk factors. In fact, the prevalences of doctor-diagnosed asthma observed in both participants and nonparticipants were almost identical to those found in a previous survey in Brussels (Michel et al. 1999). Our observations were also not distorted by a bias introduced by a possibly higher pool attendance by children with a diagnosis of asthma or from asthmatic parents because exercising in the hot and humid air of swimming pools is well tolerated by asthmatics. In the case of eNO, such bias can be formally ruled out because the association between this outcome and CPA persisted after exclusion of children with doctor-diagnosed asthma.

The importance of accurately assessing airway inflammation in the diagnosis and treatment of asthmatic patients has increasingly been acknowledged during the last few years (Silkoff et al. 2004; Skoner 2002). To our knowledge, this study is among the first to apply, in field conditions, a noninvasive test of airway inflammation as an additional outcome measure to investigate risk factors of childhood asthma. We used the eNO test, which is currently one of the best-validated indicators to monitor airway inflammation in asthmatics (Silkoff et al. 2004; Skoner 2002). Interestingly, this marker showed a pattern of associations with pool attendance that matched quite well the pattern observed with asthma, increasing also in a dose-dependent manner with the number of hours spent in pools, especially when pool attendance was during early childhood. In contrast to asthma prevalence, which correlated with pool attendance only above a certain threshold of serum IgE, eNO was positively associated with pool attendance independently of serum IgE levels. Thus, whereas asthma risk was increased only in children with elevated serum IgE, inflammatory effects associated with pool attendance, such as the effects on the pulmonary epithelium (Bernard et al. 2003; Carbonnelle et al. 2002; Lagerkvist et al. 2004), seem to concern all pool attendees.

It is improbable that these associations between asthma or lung inflammation and pool attendance are caused by swimming itself because they were unaltered by excluding children training intensively in swimming clubs. Moreover, the association observed with eNO was already statistically significant when taking into consideration the number of hours spent in a chlorinated swimming pool before 3 years of age—that is, long before children could really swim. The pool factor responsible for these effects should probably be sought among the chlorination products, which can be inhaled as gas or aerosols. One of the main culprits could be trichloramine, the gas that gives indoor pools their characteristic chlorine smell and that has recently been found to damage the lung epithelium of swimmers (Bernard et al. 2003; Carbonnelle et al. 2002; Lagerkvist et al. 2004) and to cause occupational asthma in lifeguards (Thickett et al. 2002).

Chloramines and hypochlorous acid are known to be powerful oxidants capable of rapidly disrupting tight junctions, thereby opening the main gates for the transepithelial passage of proteins into the tissues (Tatsumi and Fliss 1994). Using surfactant-associated proteins A and B as epithelial permeability markers, we have previously shown that from a level of 0.3 mg/m^3^, trichloramine in pool air induces an almost immediate increase in lung epithelium permeability (Carbonnelle et al. 2002). After repeated exposures, this gas can produce a more long-standing dose-dependent increase in lung epithelium permeability that can persist for years (Bernard et al. 2003). We think that this acute or chronic hyperpermeability caused by trichloramine in the deep lung could trigger a mechanism favoring the development of asthma in atopic subjects. Tight junctions occluding the paracellular routes represent indeed the main barrier preventing the transepithelial passage of allergens and their delivery to dendritic antigen-presenting cells (Wan et al. 1999). How this efficient barrier can be disrupted to allow the delivery of allergens is still poorly understood. Evidence from recent *in vitro* studies suggests that transepithelial delivery of allergens could be facilitated by the proteolytic activity inherent to some allergens, but it is unknown to what extent this phenomenon effectively occurs *in vivo* (Wan et al. 2001). Our data suggest that trichloramine and other chlorine-based oxidants in indoor swimming pools could play the role of chemical adjuvant, closely linked to our Western lifestyle, which would facilitate the passage of allergens across the epithelial barriers of the respiratory tract and perhaps also of other organs in contact with pool water or air. Trichloramine would be particularly active in inducing such a mechanism in the deep lung. This water-insoluble oxidant gas is indeed toxic to distal airways (Bernard et al. 2003; Carbonnelle et al. 2002), which are precisely the major sites of inflammation and airflow restriction in asthma (Tulic and Hamid 2003).

Our observations support the view that atopy and asthma, although strongly linked, are distinct components of the asthma syndrome, each with their own pattern of risk or protective factors (Von Mutius 2001). Although CPA was associated with a higher risk of developing asthma and elevated eNO, the opposite relationship was found with total serum IgE, which actually decreased with increasing CPA. We have presently no explanation for this decrease in serum IgE, which perhaps might reflect an immunotoxic action of chlorination by-products (Exon et al. 1987). Interestingly, several factors related to hygiene and infections were also found to influence specific IgE in serum. In particular, the probability of having aeroallergen-specific IgE increased with housing density and recurrent colds but decreased with the use of household chlorine, a disinfectant known to inactivate allergens (Chen and Eggleston 2001). Altogether, these positive associations between asthma or atopy and number of siblings, housing density, and recurrent colds in combination with the protective effect of chlorine bleach argue against the idea that cleanliness or reduced microbiologic exposure—the hygiene hypothesis—could by itself be responsible for the rise in childhood asthma. According to our study, the link between hygiene and childhood asthma rise would lie less in the decreasing exposure to microbiologic agents than in the increasing exposure of children to products of chlorination, the most widely used method to achieve hygiene in industrialized countries.

From a preventive point of view, the interest of the chlorine hypothesis is that it could provide an immediate course of action to reduce childhood asthma incidence because both the environment and the population at risk are identified. Our study clearly indicates that the risk of developing asthma culminates when children regularly attend an indoor chlorinated pool before 6–7 years of age. The most logical explanation for this higher sensitivity of young children, already observed in our previous study (Bernard et al. 2003), is that children cannot really swim before that age and therefore must attend the small pool, which is shallow, hot, and more heavily polluted than is the large pool. In addition, when children play or learn to swim, they also inhale and swallow more aerosols and water droplets containing hypochlorous acid and soluble chloramines. These can be carried more or less deeply into the respiratory tract depending on the size of aerosols and the respiration pattern (oral vs. nasal breathing). However, the most critical factor is that exposure to all these chlorination products culminates in children precisely at a time when their lungs are still developing. A significant proportion of lung development indeed takes place postnatally during a period that extends up to 6–8 years of age (Finkelstein and Johnston 2004). During all that time, the lung undergoes alveolarization and continued morphogenesis with a differentiation of most critical cell types and epithelial structures. It is therefore not surprising that repeated exposure of the respiratory tract to high concentrations of chlorine-based oxidants during that period can cause epithelial changes that might promote the development of asthma in sensitized subjects.

In conclusion, our study shows that chlorination by-products contaminating the air of indoor pools can act as adjuvant promoting the development of asthma in atopic children, especially in young children attending the small, heavily polluted pool. These findings further support the “pool chlorine hypothesis” suggesting that the increasing exposure of children to pool chlorine could be an important lifestyle factor implicated in the rise of childhood asthma in the developed world.

## Figures and Tables

**Table 1:** Respiratory symptoms, asthma, eNO, and serum IgE in children [no. (%)].

Indicators	Boys (*n* = 172)	Girls (*n* = 169)	*p*-Value
Respiratory symptoms			
Wheezing	20 (11.6)	12 (7.1)	0.157
Chest tightness	9 (5.2)	10 (5.9)	0.770
Shortness of breath	12 (7.0)	12 (7.1)	0.964
Cough	35 (20.3)	27 (16.0)	0.307
Total serum IgE			
kIU/L (range)	63.3 (6.7–3,204)	69.7 (4.0–3,545)	0.523
> 100 kIU/L	57 (32.9)	60 (35.5)	0.679
Aeroallergen-specific serum IgE			
Panel of 12 aeroallergens	61 (35.3)	47 (27.8)	0.148
House-dust mite	40 (23.1)	24 (14.2)	0.037
Dog	6 (3.4)	6 (3.6)	0.950
Cat	14 (8.1)	5 (4.7)	0.041
Pollen	21 (12.1)	17 (10.1)	0.567
eNO			
Geometric mean [range (ppb)]	11.0 (2.8–91.6)	10.2 (3.2–101)	0.290
> 30 ppb	16 (9.2)	13 (7.7)	0.606
> 30 ppb (without doctor-diagnosed asthma)	8 (4.6)	11 (6.5)	0.447
Asthma			
Doctor diagnosed	18 (10.4)	10 (5.9)	0.065
Screened with the EIB test	6 (3.4)	6 (3.6)	0.736
Total asthma (diagnosed and/or positive EIB test)	24 (14.0)	16 (9.5)	0.198
Doctor-diagnosed asthma and/or eNO > 30 ppb	26 (15.1)	18 (10.7)	0.219
Total asthma and/or eNO > 30 ppb	32 (21.9)	24 (16.5)	0.273

**Table 2:** Predictors of asthma and elevated eNO in children.

Indicator	Predictors	OR^a^ (95% CI)	*p*-Value
Doctor-diagnosed asthma	Mother and/or father with asthma	4.01 (1.68–9.55)	0.0017
	Aeroallergen-specific serum IgE	3.53 (1.57–7.95)	0.0024
	Sex (boy)	2.65 (1.11–6.32)	0.0284
	CPA (100 hr)	1.14 (0.99–1.31)	0.0691
	Pets from birth	2.33 (0.93–5.85)	0.0725
	No. of siblings	1.32 (0.97–1.81)	0.0815
Total asthma (doctor diagnosed and/or screened with EIB test)	Aeroallergen-specific serum IgE	2.76 (1.28–5.96)	0.0095
	Total serum IgE (100 kIU/L)	1.09 (1.02–1.16)	0.0131
	Mother and/or father with asthma	2.40 (1.08–5.31)	0.0310
	Housing density (persons/room)	3.30 (1.11–9.87)	0.0320
	CPA (100 hr)	1.15 (0.99–1.31)	0.0512
	Pets from birth	2.18 (0.94–5.04)	0.0680
	Household chlorine bleach	0.46 (0.19–1.15)	0.0960
eNO > 30 ppb	Aeroallergen-specific serum IgE	21.1 (5.37–82.7)	< 0.0001
	CPA (100 hr)	1.30 (1.10–1.43)	0.0027
	Total serum IgE (100 kIU/L)	1.10 (1.02–1.19)	0.0155
	BMI (kg/m^2^)	0.77 (0.63–0.95)	0.0156
	Pets during the previous 2 years	0.22 (0.06–0.80)	0.0216
	Day nursery attendance	0.32 (0.11–0.91)	0.0329
	Mother and/or father with hay fever	2.73 (1.02–7.30)	0.0461
	Housing density (persons/room)	2.69 (0.97–7.51)	0.0584
eNO > 30 ppb without doctor-diagnosed asthma	Aeroallergen-specific serum IgE	11.5 (2.57–51.5)	0.0014
	Total serum IgE (100 kIU/L)	1.09 (1.01–1.17)	0.0255
	Pets during the previous 2 years	0.10 (0.01–0.83)	0.0335
	Housing density (persons/room)	4.55 (1.09–18.9)	0.0372
	CPA (100 hr)	1.22 (1.00–1.50)	0.0490
	Mother and/or father with hay fever	2.95 (0.97–9.00)	0.0570
eNO > 30 ppb and/or doctor-diagnosed asthma	Aeroallergen-specific serum IgE	5.49 (2.66–11.3)	< 0.0001
	CPA (100 hr)	1.19 (1.02–1.37)	0.0135
	No. of siblings	1.33 (1.03–1.71)	0.0273
	Mother and/or father with hay fever	2.11 (1.03–4.32)	0.0422
	Mother and/or father with asthma	2.06 (0.90–4.69)	0.0863
eNO > 30 ppb and/or total asthma	Aeroallergen-specific serum IgE	4.11 (2.06–8.20)	< 0.0001
	Mother and/or father with hay fever	2.65 (1.37–5.11)	0.0038
	Total serum IgE (100 kIU/L)	1.09 (1.02–1.16)	0.0128
	CPA (100 hr)	1.17 (1.02–1.33)	0.0215
	Housing density (persons/room)	3.09 (1.15–8.26)	0.0247
	No. of siblings	1.24 (0.98–1.57)	0.0757

CI, confidence interval.

a ORs calculated by multiple logistic regression analysis of 17 independent variables (detailed in “Materials and Methods”). The table lists only those associations emerging with a *p* < 0.1.

**Table 3:** Risk of asthma and/or elevated eNO with CPA [OR (95% confidence interval)] in nonatopic and atopic children on the basis of total and aeroallergen-specific serum IgE.

	Total serum IgE		Total serum IgE		Aeroallergen-specific serum IgE		Total serum IgE > 100 kIU/L and/or aeroallergen-specific serum IgE	
Indicator	< 56 kIU/L (*n* = 171)	> 56 kIU/L (*n* = 171)	< 100 kIU/L (*n* = 225)	> 100 kIU/L (*n* = 117)	No (*n* = 234)	Yes (*n* = 108)	No (*n* = 181)	Yes (*n* = 161)
Doctor-diagnosed asthma	1.12 (0.89–1.42) *p* = 0.347	1.34 (1.03–1.75) *p* = 0.032	1.03 (0.81–1.31) *p* = 0.81	1.57 (1.07–2.30) *p* = 0.020	1.12 (0.91–1.39) *p* = 0.28	1.25 (0.99–1.57) *p* = 0.054	0.99 (0.78–1.37) *p* = 0.81	1.29 (1.05–1.59) *p* = 0.014
Total asthma (doctor diagnosed and/or screened with EIB test)	1.11 (0.85–1.45) *p* = 0.447	1.42 (1.06–1.91) *p* = 0.019	0.98 (0.76–1.27) *p* = 0.89	1.79 (1.066–2.72) *p* = 0.007	1.07 (0.86–1.33) *p* = 0.53	1.20 (0.97–1.47) *p* = 0.09	0.99 (0.73–1.37) *p* = 0.97	1.27 (1.04–1.56) *p* = 0.022
eNO > 30 ppb	1.40 (0.99–1.96) *p* = 0.056	1.33 (1.03–1.72) *p* = 0.031	1.65 (1.19–2.28) *p* = 0.003	1.19 (0.86–1.64) *p* = 0.28	1.87 (1.07–3.29) *p* = 0.029	1.23 (0.96–1.58) *p* = 0.09	1.91 (1.08–3.37) *p* = 0.026	1.20 (0.94–1.53) *p* = 0.14
eNO > 30 ppb, no diagnosed asthma	1.42 (0.83–2.43) *p* = 0.20	1.27 (0.96–1.67) *p* = 0.089	1.62 (1.08–2.44) *p* = 0.02	1.07 (0.73 –1.58) *p* = 0.73	1.71 (0.97–3.03) *p* = 0.066	1.06 (0.78–1.45) *p* = 0.71	1.78 (0.99–3.20) *p* = 0.053	1.07 (0.79–1.46) *p* = 0.64
eNO > 30 ppb and/or doctor-diagnosed asthma	1.25 (1.04–1.51) *p* = 0.019	1.38 (1.01–1.88) *p* = 0.043	1.20 (1.02–1.42) *p* = 0.029	1.66 (1.11–2.49) *p* = 0.014	1.18 (1.01–1.38) *p* = 0.04	1.16 (0.94–1.43) *p* = 0.17	1.27 (1.04–1.55) *p* = 0.017	1.16 (0.97–1.40) *p* = 0.11
eNO > 30 ppb and/or total asthma	1.13 (0.92–1.38) *p* = 0.25	1.49 (1.09–2.04) *p* = 0.012	1.12 (0.97–1.31) *p* = 0.14	1.71 (1.13–2.61) *p* = 0.012	1.18 (1.01–1.38) *p* = 0.04	1.16 (0.94–1.43) *p* = 0.17	1.14 (0.97–1.35) *p* = 0.12	1.21 (0.99–1.48) *p* = 0.055

Adjusted ORs calculated for each 100 hr CPA and adjusted for covariates listed in Table 2.
